# Supplementary material for: Characterization and significance of extracellular polymeric substances, reactive oxygen species, and extracellular electron transfer in methanogenic biocathode
Source: Sci Rep. 2021 Apr 12;11:7933. doi: 10.1038/s41598-021-87118-w (PMC8041852; doi:10.1038/s41598-021-87118-w)
Supplement: Supplementary file 1 — Supplementary Informations. [file 41598_2021_87118_MOESM1_ESM.docx]

**Supplementary Information**

**Characterization and significance of extracellular polymeric substances, reactive oxygen species, and extracellular electron transfer in methanogenic biocathode**

Basem S. Zakaria^1^, Bipro Ranjan Dhar^1,*^

^1^Civil and Environmental Engineering, University of Alberta, 9211-116 Street NW, Edmonton, AB, Canada T6G 1H9

^*^Corresponding author: Bipro Ranjan Dhar (bipro@ualberta.ca)

Number of pages: 22

Number of figures: 9

Number of tables: 3

Number of text modules: 6

**Text S1.** **Estimation of specific surface area of electrodes**

**1. Stainless-steel mesh electrode**

The surface area was calculated according to Zhang et al. ^1^ with minor modification.

$$S=2\pi bdn(n+1)+\frac{3}{2}\pi d^{2}\left( n+1 \right)^{2}$$

Where, (b) is the pore size, and (d) is the wire diameter, (n) is 70 mesh

b= 0.02 cm, d = 0.016 cm

The specific surface area based on reactor volume = 0.0016/0.00038 m^2^/m^3^ = 4.23 m^2^/m^3^

**2. Carbon fiber electrode**

The surface area of carbon fiber was calculated using two approaches previously reported in the literature ^2,3^:

1. Considering every single filament in the carbon fiber bundle (filament bundle consists of 24000 single filaments, each with diameter 7 µm, and the length = 130 cm)

S.A. = 2пrh x n = 2 x 3.14 x 0.0007 cm x 130 cm x 24000 = 13715.5 cm^2^

The specific surface area based on reactor volume = 1.372 m^2^ / 0.00038 m^3^ = 3609 m^2^/m^3^

2. Considering all filaments in a bundle act together as a single fiber (width = 0.5 cm, length = 130 cm, height = 0.1 cm).

S.A.= 2 (wh +Lw + Lh) = 156.4 cm^2^

The specific surface area based on reactor volume = 0.0156 m^2^ / 0.00038 m^3^ = 41 m^2^/m^3^


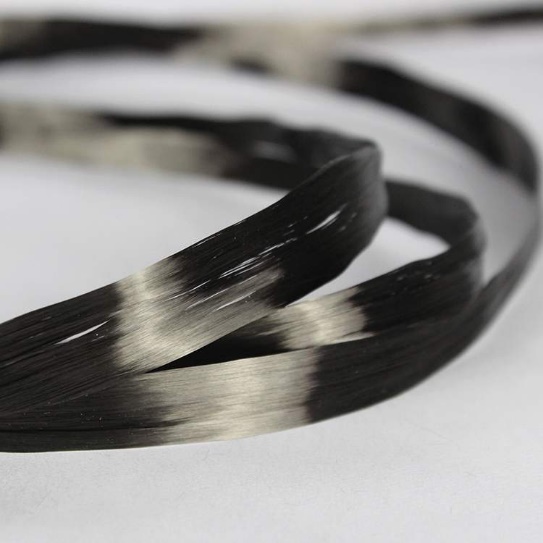


W

h

**Fig. S1.** Photograph of carbon fiber bundle.

**Text S2.** **EPS extraction protocol and analytical methods for EPS measurement**

**EPS Extraction:** CER method for EPS extraction was performed according to Frølund et al. ^4^ with some minor modification, 52 cm^2^ of carbon fiber and 0.054 cm^2^ of stainless-steel were resuspended in 0.1 M PBS buffer, pH 7.4. Then, 2 g CER (Dowex Marathon C sodium form, Sigma-Aldrich, USA), washed with 0.1 M PBS for 15 min (10 mL g^-1^ Dowex), was added to each sample. The samples were agitated at a high capacity for 20 min on the shaker (Vortex Mixer, Fisher Scientific, USA). The heating method was conducted according to Xu et al. ^5^, the electrodes were washed twice with 0.9% NaCl (w/v), then resuspended in 0.9% NaCl (w/v). The samples were heated at 60^o^C in a water bath for 30 minutes. For both methods, the EPS were harvested by centrifugation three times at 20,000 × g for 20 minutes at 4^o^C. The supernatant was collected and filtered using a 0.2 µm filter and the EPS stored at -20^o^C for further analysis. Also, the pellets were collected to examine the cell lysis using Glucose-6-Phosphate Dehydrogenase kit (Sigma-Aldrich, USA). EPS levels were presented as the mass of EPS per unit surface area of the electrode (mg/cm^2^) for comparing EPS levels in two reactors.

**Analytical methods for EPS measurement:** The total carbohydrate content was measured using a phenol-sulfuric acid method using glucose standards according to DuBois et al. ^6^ with some minor modification. 2 mL of EPS was mixed with 5 mL of concentrated sulfuric acid followed by the addition of 0.05 mL of 80% wt. phenol. Then, the samples were incubated at room temperature for 10 minutes, then shake followed by incubation for 20 minutes at 30^o^C. The samples were measured at 490 nm using UV-spectrophotometer (Model DR 3900, HACH, Germany). The protein contents of the EPS were measured using Pierce Modified Lowry Protein Assay Kit (Thermo Fisher, USA) according to the manufacturer’s instructions. Uronic acid was measured according to Blumenkrantz and Asboe-Hansen ^7^ and Ghods et al. ^8^ with glucuronic acid (Sigma-Aldrich, USA) as a standard. Heme contents were measured using the Heme Assay Kit (Heme Assay Kit, Sigma-Aldrich, USA) according to the manufacturer’s instructions. The extracellular DNA (eDNA) was quantified using the diphenylamine colorimetric method with calf thymus DNA as the standard ^9^. 100 µL EPS was pipetted in a cuvette and 2.5 mL of the reagent (0.2 ppm DAPI (4, 6-diaminodino-2-phenylindole) in 100 mM NaC1, l0 mM EDTA, 10 mM PBS, pH 7.2) was added. All the above measurements were conducted in triplicate.

| **Table S1.** EPS composition using CER and heating methods. | | | | | |
| --- | --- | --- | --- | --- | --- |
|  | **Carbohydrate**  **(mg/cm^2^)** | **Proteins**  **(mg/cm^2^)** | **Heme**  **(mg/cm^2^)** | **Uronic Acid**  **(mg/cm^2^)** | **eDNA**  **(mg/cm^2^)** |
| **CER Method** | | | | | |
| **Anode (CF-CF)** | 28.225 | 133.545 | 40.58 | 10.6 | 0.55 |
| **Cathode (CF-CF)** | 25.775 | 170.025 | 16.52 | 6.2 | 0.39 |
| **Anode (CF-SS)** | 34.775 | 146.405 | 37.94 | 11.665 | 0.685 |
| **Cathode (CF-SS)** | 52.14 | 212.79 | 34.22 | 15 | 1.525 |
| **Heating Method** | | | | | |
| **Anode (CF-CF)** | 35.575 | 109.29 | 50.45 | 13.065 | 0.69 |
| **Cathode (CF-CF)** | 27.78 | 163.29 | 18.62 | 8.27 | 0.365 |
| **Anode (CF-SS)** | 24.1 | 172.855 | 41.13 | 11.32 | 0.46 |
| **Cathode (CF-SS)** | 52.61 | 223.885 | 37.57 | 15.2 | 1.715 |

**Text 3. Method for CLSM imaging**

Confocal laser scanning microscopy (CLSM) was used to visualize and examine the EPS structure on the electrodes. Portions of carbon fibers and stainless-steel mesh were cut with an aseptic scissor from different locations and washed with 0.1 M PBS buffer (pH 7.4) to remove any debris. The electrodes were fluorescently stained with TOTO-1 (Thermo Fisher, USA) and Concanavalin A (ConA) Alexa Fluor 633 Conjugate (Thermo Fisher, USA) for one hour in the dark for eDNA and EPS visualization, respectively. Then, the samples were washed again with PBS buffer to remove any non-specific binding stains. The stained electrodes were placed on MatTek dishes with a 1.5 coverslip (MatTek co., USA) to ensure that the biofilm was not compressed. The visualization of the electrodes was performed using a Leica inverted DMI 6000 B microscope (Leica Microsystems, USA). CLSM was equipped with an argon laser and helium-neon lasers. The images were acquired using x63/1.3 water immersion lens using LAS AF software (Leica Microsystems, USA, https://www.leica-microsystems.com/) at a resolution of 1024 x 1024 with 120 nm pixel size. Images z-stack were acquired with several steps 29 with z-step size 0.29 µm with smart grain 687. The quantitative analysis of EPS structure was carried out using biofilm image processing COMSTAT software (COMSTAT2, Version 2.1, Dk, http://www.comstat.dk/)^10–12^. The EPS intensities were reported as intensity/um^3^ of the electrode.


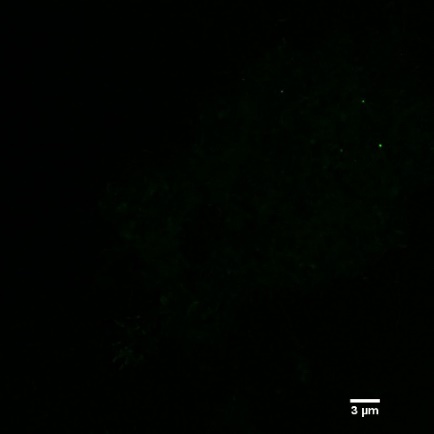

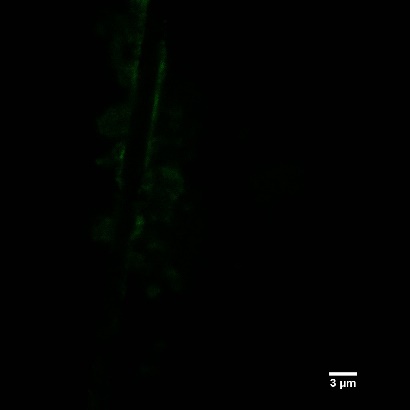

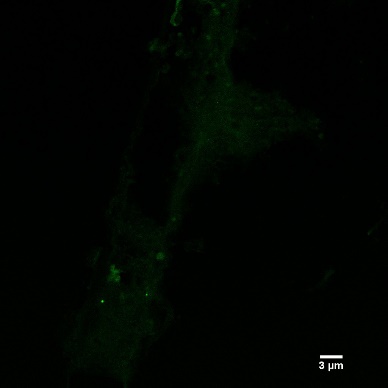

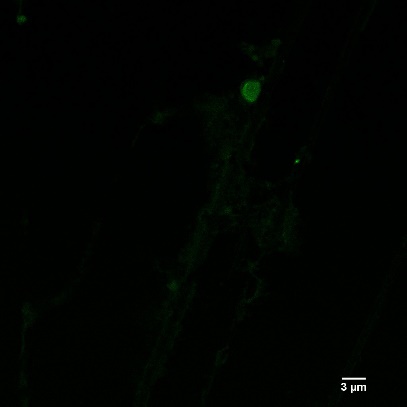


(a)

(b)

(c)

(d)

**Fig. S2.** CLSM of reactive oxygen species (ROS). Anode (CF-CF) (a), cathode (CF-CF) (b), anode (CF-SS) (c), and cathode (CF-SS) (d). (This figure has been analyzed using COMSTAT2, Version 2.1, Dk, http://www.comstat.dk/).

**Text S4. Methods for measurement of EET gene expression levels.** The concept is to design primers to detect most of the *omc*B, *omc*C, *omc*E, *omc*Z, *omc*S, and *pil*A gene sequences as possible. The primer design was performed similarly to Lin et al. ^13^, and *rec*A housekeeping gene was used as a reference ^14^. The genome sequences of the anode biofilm were collected from the National Center for Biotechnology Information (NCBI) (https://www.ncbi.nlm.nih.gov). These followed by multiple-aligned using the ClustalX alignment tool (ClustalX, Version 2.1, http://www.clustal.org/clustal2/) to select most of the homologous gene regions ^15^. Then, gene-specific primers for RT-PCR were designed with Primer3 software (Primer3, Version 4.1.0, http://bioinfo.ut.ee/primer3) by selecting an almost similar range of melting temperatures. The Basic Local Alignment Search Tool (BLAST) (https://blast.ncbi.nlm.nih.gov/Blast.cgi) was used to check the specificity of the primers. Also, the primers were experimentally examined using agarose gel electrophoresis. The primers were prepared by Integrated DNA Technologies (IDT, USA) and are listed in Table S1.

Total RNA was extracted using RNeasy PowerSoil Total RNA Kit (Qiagen, CA), then the purity and concentration were examined using Nanodrop (2000C, Thermo Scientific, USA). Subsequently, cDNA synthesis was performed using QuantiTect Reverse Transcription Kit (Qiagen, CA). Then, RT-PCR mixtures were prepared in 25 uL reactions using QuantiFast SYBR Green PCR Kit (Qiagen, CA) as the following: 1 µL of the template, 12.5 µL 2x master mix, 2.5 µL forward and reverse specific primer, and 6.5 µL nuclease-free water. CFX 96 real-time PCR system with a C1000 Thermal Cycler (Bio-Rad, USA) was used with the following cycling conditions according to the QuantiFast SYBR Green PCR Kit’s protocol; PCR initial heat activation cycle at 95 ˚C for 5 min, 35 cycles at 95 ˚C for 10 sec and 60 ˚C for 30 sec, and finally, one cycle at 40 ˚C for 30 seconds. Triplicate reactions were run for all samples.

| **Table S2.** Primers used for studying gene expression levels | | |
| --- | --- | --- |
|  | Forward (5’-3’) | Reverse (5’-3’) |
| *rec*A | CACCGGCATAATCTCCAAGT | ATCTTGCGGATATCGAGACG |
| *pil*A | TTATGATCGTGGTTGCCATT | CCGCAGTTAAACCTTCTGCT |
|  | GAGCGATGTTCTTTCCGTTT | AATCCTGATAGGCGGGAATC |
| omcB | CCGAAAATTACGCAGGTGTT | GGAGTTCACGAAACCAAGGA |
|  | GAGGGACGATGTCAACCTGT | TTCGCAAGGTAGCTGTTGTG |
| omcC | CTGGTACTCTGGGTGGCATT | GCTGTGGTTAGCAGCATTGA |
|  | ACGAGTTCCAGACCAACACC | GGCAGTATCGTCCCAGTTGT |
| omcE | ATGTCCTACGGCGATGCTAT | GTAACCTGCAGGAAGGTGGA |
|  | CTCGTCCAGCAGCATGAATA | GGGGTGATCATTGCTCAGAT |
| omcZ | GGACGTATTGTGGCAGAGGT | GGCCACTACATTCCGACCTA |
|  | GGCCACTACATTCCGACCTA | GGACGTATTGTGGCAGAGGT |
| omcS | GCTGACTACACCGTGCTCAA | TGAACTCGTATGCCAGGTTG |
|  | GGCAAGTACCGTCGTTTTGT | GTAGCTTCCGTCCGGTTGTA |

**Text S5. Microbial quantification using RT-PCR.** qPCR was performed to quantify bacterial cell numbers before and after AgNPs injection. 16S universal primers, 357Wf: CCTACGGGNGGCWGCAG and 785R: GACTACHVGGGTATCTAATCC, were used to quantify the DNA samples. qPCR mixtures were prepared in 25 uL reactions using QuantiFast SYBR Green PCR Kit (Qiagen, CA) as the following: 2 µL of the DNA template, 12.5 µL 2x master mix, 2.5 µL forward and reverse specific primer, and 5.5 µL nuclease-free water. CFX 96 real-time PCR system with a C1000 Thermal Cycler (Bio-Rad, USA) was used with the following cycling conditions according to the QuantiFast SYBR Green PCR Kit’s protocol; PCR initial heat activation cycle at 95 ˚C for 5 min, 35 cycles at 95 ˚C for 10 sec and 60 ˚C for 30 sec, and finally, one cycle at 40 ˚C for 30 seconds. Triplicate reactions were run for all samples.

**Fig. S3.** Volumetric current densities from CF-CF and CF-SS reactors. Note. Volumetric current densities indicate the current normalized by the total working volume of the reactor. The results from 3 representative batch cycles during steady-state are shown here. The error bars indicate the standard deviation of three replicates (n = 3).

**Text S6.** **Electrochemical impedance spectroscopy (EIS)**

EIS was performed with a multi-channel VSP potentiostat (VSP, Bio-Logic Science Instruments, France), and the data was recorded using EC-Lab software (EC-Lab, Version 10.38, BioLogic Science Instruments, France, https://www.biologic.net/). EIS measurements performed using two reactor configurations; two-electrode configuration for the whole cell (anode as working electrode and cathode and reference electrodes as counter electrodes), and three-electrode configuration to test anode and cathode electrodes separately, where anode/cathode as working electrode and the other as counter electrode, and Ag/AgCl was used as a reference electrode ^16–18^. The potentials set depending on the potential of anode and cathode electrodes. The sinusoidal amplitude was fixed at 1 mV with a frequency range of 100 kHz to 0.01 Hz. Then, the data were fitted using Z-fit with an equivalent circuit model (ECM), as previously described in the literature ^18^. Equivalent circuit model provided as following; [Rs (ohmic resistance)] [Rct (activation resistance) Q1 (phase element)] [Rd (concentration resistance) Q2 (phase element)], was used for the whole cell, and [Rs (ohmic resistance)] [(Rct (activation resistance) Q1 (phase element)] Rs (ohmic resistance) [Rd (concentration resistance) Q2 (phase element)] ECM was used for testing the individual electrodes.


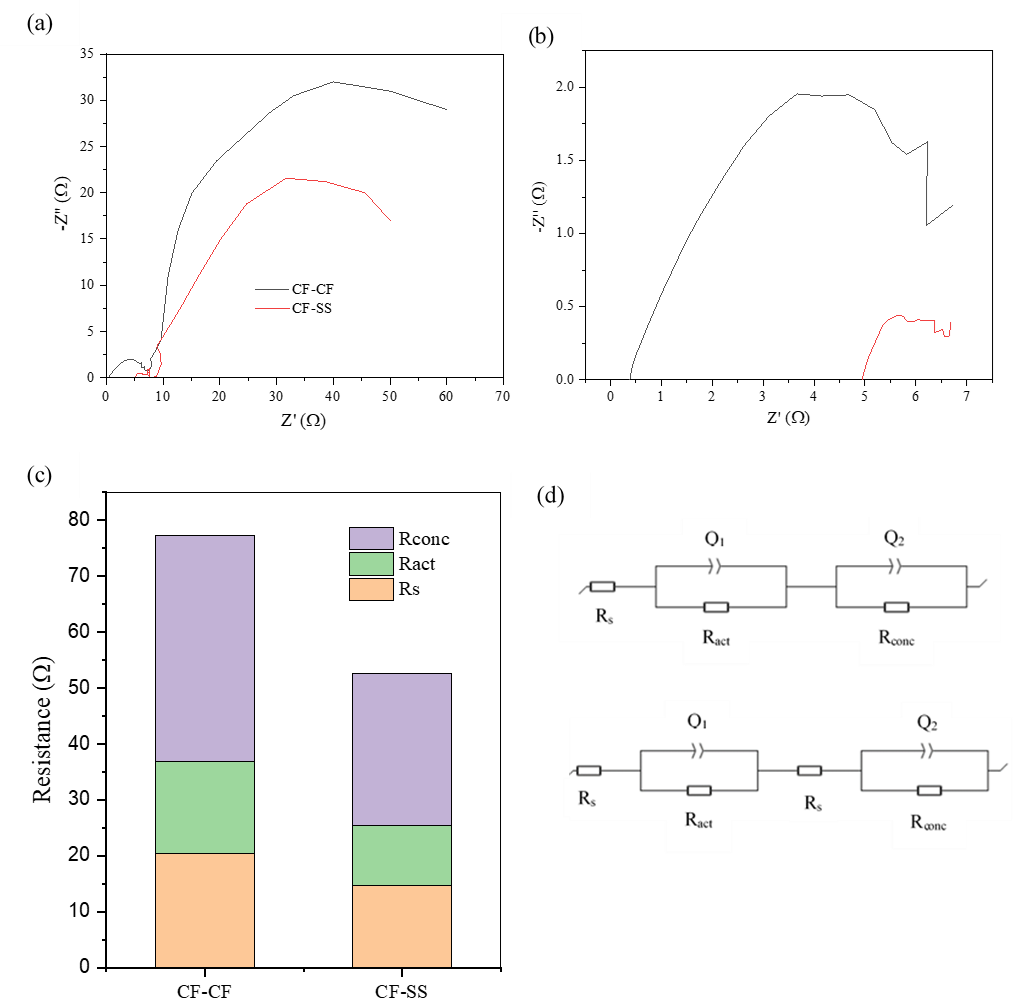


**Fig. S4.** Nyquist plot for whole cell (a), the enlarged Nyquist plot for the highest frequency region (b), internal resistances (c), equivalent circuit models (d) of CF-CF and CF-SS reactors.


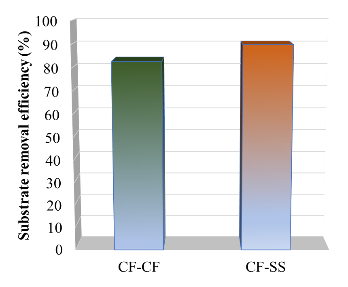


(a)

(b)

(c)

**Fig. S5.** COD concentration (a), and VFA profiles (b, c) in CF-CF and CF-SS reactors. The error bars show the standard deviation of three replicates (n = 3).


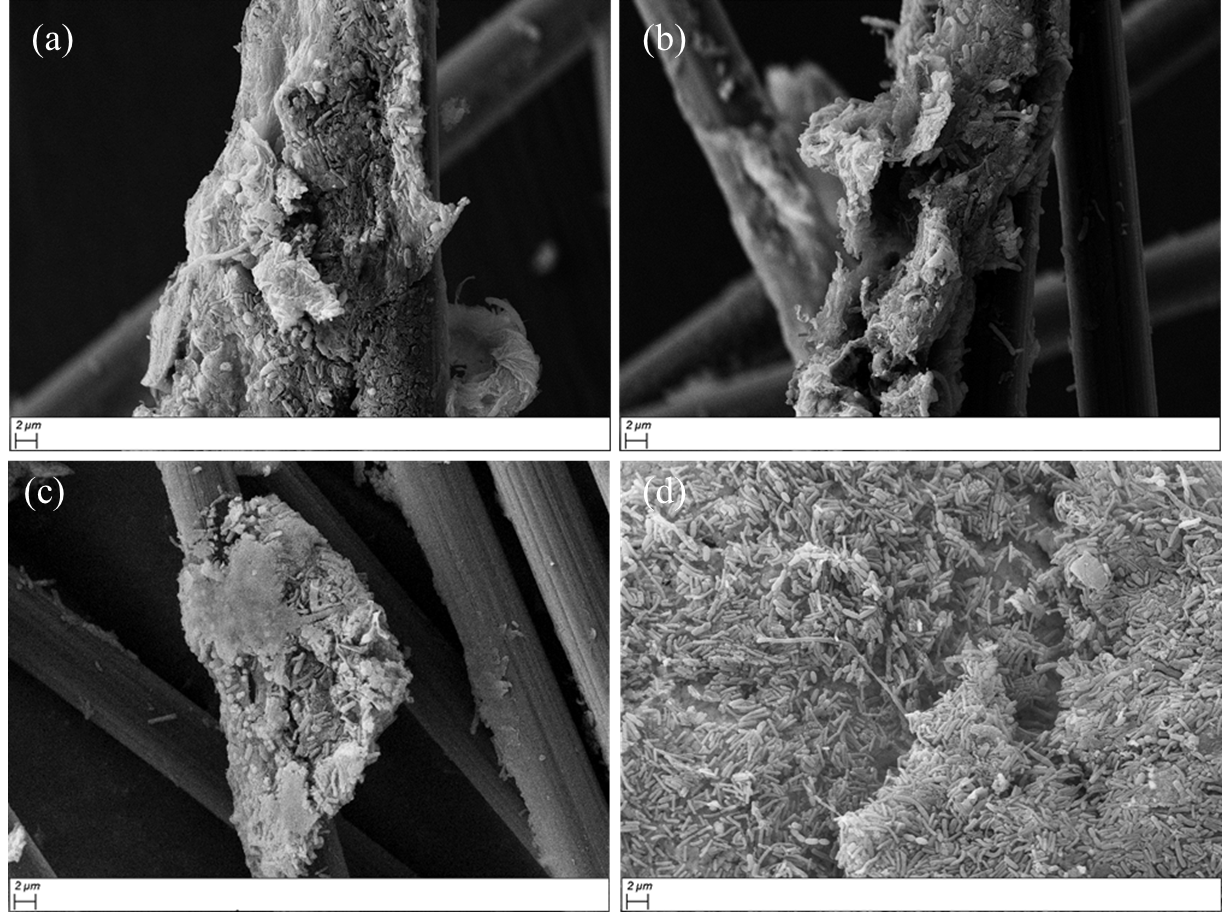


**Fig. S6.** Representative SEM images of biofilms developed on anode (CF-CF) (a), cathode (CF-CF) (b), anode (CF-SS) (c), and cathode (CF-SS) (d).


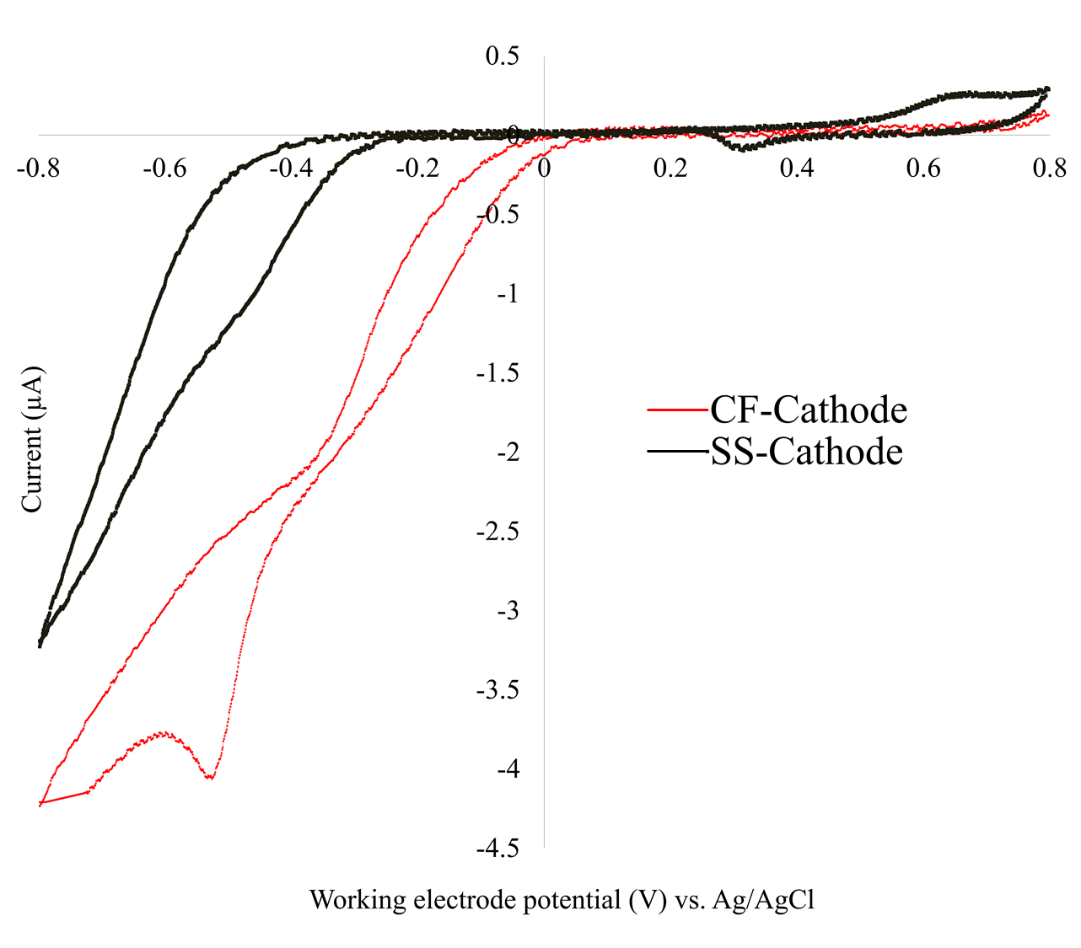


**Fig. S7.** Cyclic voltammetry of EPS extracted from biocathode in CF-CF and CF-SS reactors.

| **Table S3.** The diversity and richness of the biofilms of both systems. | | | | | | | |
| --- | --- | --- | --- | --- | --- | --- | --- |
| **Reactor/electrode** | | **Chao 1** | **Phylogenetic distance** | **OTUs** | **Pielou's evenness** | **Shannon** | **Coverage** |
| **CF-CF** | **Anode** | 137 | 11.68 | 132 | 0.45 | 3.14 | 1.0 |
|  | **Cathode** | 76 | 7.19 | 76 | 0.63 | 3.95 | 1.0 |
| **CF-SS** | **Anode** | 146 | 13.57 | 146 | 0.72 | 5.18 | 1.0 |
|  | **Cathode** | 155 | 12.32 | 150 | 0.71 | 5.10 | 1.0 |

(a)

(b)

**Fig. S8.** Relative abundance of microbial community. Bacterial primer phylum level (a), and archaeal primer phylum level (b).

(a)

(b)

**Fig. S9.** Principal Component Analysis of the bacterial (a) and archaeal (b) communities in biocathode of CF-CF and CF-SS reactors.

**References**

1. Zhang, Y., Merrill, M. D. & Logan, B. E. The use and optimization of stainless steel mesh cathodes in microbial electrolysis cells. *Int. J. Hydrogen Energy* **35**, 12020–12028 (2010).

2. Brunschweiger, S. *et al.* The effect of clogging on the long-term stability of different carbon fiber brushes in microbial fuel cells for brewery wastewater treatment. *Bioresour. Technol. Reports* **11**, 100420 (2020).

3. Lanas, V. & Logan, B. E. Evaluation of multi-brush anode systems in microbial fuel cells. *Bioresour. Technol.* **148**, 379–385 (2013).

4. Frølund, B., Palmgren, R., Keiding, K. & Nielsen, P. H. Extraction of extracellular polymers from activated sludge using a cation exchange resin. *Water Res.* **30**, 1749–1758 (1996).

5. Xu, J., Sheng, G. P., Ma, Y., Wang, L. F. & Yu, H. Q. Roles of extracellular polymeric substances (EPS) in the migration and removal of sulfamethazine in activated sludge system. *Water Res.* **47**, 5298–5306 (2013).

6. DuBois, M., Gilles, K. A., Hamilton, J. K., Rebers, P. A. & Smith, F. Colorimetric method for determination of sugars and related substances. *Anal. Chem.* **28**, 350–356 (1956).

7. Blumenkrantz, N. & Asboe-Hansen, G. New method for quantitative determination of uronic acids. *Anal. Biochem.* **54**, 484–489 (1973).

8. Ghods, S., Sims, I. M., Moradali, M. F. & Rehma, B. H. A. Bactericidal compounds controlling growth of the plant pathogen pseudomonas syringae pv. actinidiae, which forms biofilms composed of a novel exopolysaccharide. *Appl. Environ. Microbiol.* **81**, 4026–4036 (2015).

9. Brunk, C. F., Jones, K. C. & James, T. W. Assay for nanogram quantities of DNA in cellular homogenates. *Anal. Biochem.* **92**, 497–500 (1979).

10. Comstat 2. http://www.comstat.dk/.

11. Heydorn, A. *et al.* Quantification of biofilm structures by the novel computer program COMSTAT. *Microbiology* **146**, 2395–2407 (2000).

12. Vorregaard, M. Comstat2 - a modern 3D image analysis environment for biofilms. (2008).

13. Lin, L., Chowdhury, B., Zakaria, B. S. & Dhar, B. R. Temperature-dependent (20–55 °C) electrocatalytic characteristics during ethanol/propionate degradation by methanogenic communities grown on conductive carbon fibers. *Chem. Eng. J.* 123566 (2019) doi:10.1016/j.cej.2019.123566.

14. Rivas, M., Seeger, M., Holmes, D. S. & Jedlicki, E. A Lux-like quorum sensing system in the extreme acidophile Acidithiobacillus ferrooxidans. *Biol. Res.* **38**, 283–297 (2005).

15. Thompson, J. The CLUSTAL_X windows interface: flexible strategies for multiple sequence alignment aided by quality analysis tools. *Nucleic Acids Res.* **25**, 4876–4882 (1997).

16. Xochitl, D. B., Sevda, S., Vanbroekhoven, K. & Pant, D. The accurate use of impedance analysis for the study of microbial electrochemical systems. *Chem. Soc. Rev.* **41**, 7228–7246 (2012).

17. Hidalgo, D., Sacco, A., Hernández, S. & Tommasi, T. Electrochemical and impedance characterization of Microbial Fuel Cells based on 2D and 3D anodic electrodes working with seawater microorganisms under continuous operation. *Bioresour. Technol.* **195**, 139–146 (2015).

18. Wang, B., Liu, W., Zhang, Y. & Wang, A. Bioenergy recovery from wastewater accelerated by solar power: Intermittent electro-driving regulation and capacitive storage in biomass. *Water Res.* **175**, 115696 (2020).
